# A Case of Cochlear Implantation in a Deaf Patient With Eosinophilic Otitis Media in Whom Post-implantation Hearing Threshold Improved After Introduction of Dupilumab

**DOI:** 10.7759/cureus.65059

**Published:** 2024-07-21

**Authors:** Atsushi Fukuda, Kimiko Hoshino, Shinya Morita, Keishi Fujiwara, Akihiro Homma

**Affiliations:** 1 Department of Otolaryngology-Head and Neck Surgery, Faculty of Medicine and Graduate School of Medicine, Hokkaido University, Sapporo, JPN

**Keywords:** biologics, dupilumab, cochlear implant, cochlear implantation, eosinophilic otitis media

## Abstract

Eosinophilic otitis media (EOM) is a rare, intractable, and chronic form of otitis media. The associated hearing loss often progresses to deafness, necessitating cochlear implantation (CI). EOM is associated with type 2 inflammatory conditions such as asthma and chronic rhinosinusitis with nasal polyps (CRSwNP). Dupilumab, a monoclonal antibody targeting the IL4Rα subunit, has shown efficacy in mitigating type 2 inflammatory diseases, including asthma and CRSwNP. Recent studies also suggest its effectiveness in treating EOM. We report a unique case of CI for EOM, in which the post-implant hearing threshold improved after the introduction of dupilumab. The patient was a 63-year-old man with a history of asthma and multiple nasal polypectomies. Eosinophils were detected in otorrhea samples from both ears, leading to an EOM diagnosis four years prior. Despite local corticosteroid therapy, his hearing gradually deteriorated. One month ago, he experienced sudden bilateral deafness and was referred to our hospital. The right tympanic membrane had a pinhole perforation but no otorrhea. CT showed a small amount of soft tissue density in the right middle ear, while the left side displayed cochlear osteolysis with soft tissue density. A right CI and myringoplasty were performed one and a half months after his visit. The cochleostomy revealed no perilymph leakage, confirming that the scala tympani was filled with granulation tissue. The electrode was inserted successfully despite the granulation, and all electrodes were placed correctly. Six months after CI, his hearing threshold with the cochlear implant remained poor at 67.5 dB. However, upon starting dupilumab therapy seven months postoperatively, his hearing threshold with the cochlear implant rapidly improved to 31.3 dB. Intraoperative findings suggested that the scala tympani was filled with granulation tissue, indicating significant cochlear inflammation due to EOM. The subsequent hearing improvement after introducing dupilumab may be attributed to the reduction or disappearance of granulation in the cochlea, allowing for effective electrical stimulation from the electrodes to the spiral ganglion. This case suggests the potential for improved postoperative hearing outcomes in CI for EOM when inflammation is effectively controlled with dupilumab.

## Introduction

Eosinophilic otitis media (EOM) is a rare, intractable, and chronic form of otitis media characterized by high-viscosity otorrhea [1]. Local and systemic administration of corticosteroids is considered effective, but remission is very difficult to achieve, often resulting in progressive hearing loss and deafness [2]. EOM is considered a type 2 inflammatory disease along with asthma and chronic rhinosinusitis with nasal polyps (CRSwNP). In fact, many cases of EOM are associated with these conditions [3]. Dupilumab, a fully human immunoglobulin G (IgG) 4 monoclonal antibody, targets the interleukin (IL) 4 receptor α (IL4Rα) subunit, thereby inhibiting IL4 and IL13, the major drivers of human type 2 inflammatory disease [4]. Numerous reports have demonstrated the efficacy of dupilumab in asthma and CRSwNP [4,5]. Recently, the efficacy of dupilumab in EOM has also been documented [3,6,7].

Cochlear implantation (CI) is indicated for EOM patients who have become deaf. Although several studies have reported favorable hearing outcomes with CI in EOM patients, the optimal surgical technique and its effectiveness have not yet been established [8-12]. This report describes a case of EOM in which the patient initially had poor postoperative hearing after CI but showed improved hearing following the introduction of dupilumab. This article was previously presented as a meeting abstract at the 7th Congress of European ORL-HNS on June 15-19, 2024.

## Case presentation

The patient was a 63-year-old man with a history of asthma and multiple nasal polypectomies. Four years ago, eosinophils were detected in both ears' otorrhea, and intratympanic topical corticosteroid administration was started with the diagnosis of EOM. However, his hearing gradually deteriorated. One month ago, he developed fever and vertigo, and suddenly became bilaterally deaf. Systemic corticosteroid administration for one week did not improve his hearing. Therefore, he was referred to our hospital as a candidate for CI.

**Figure 1:**
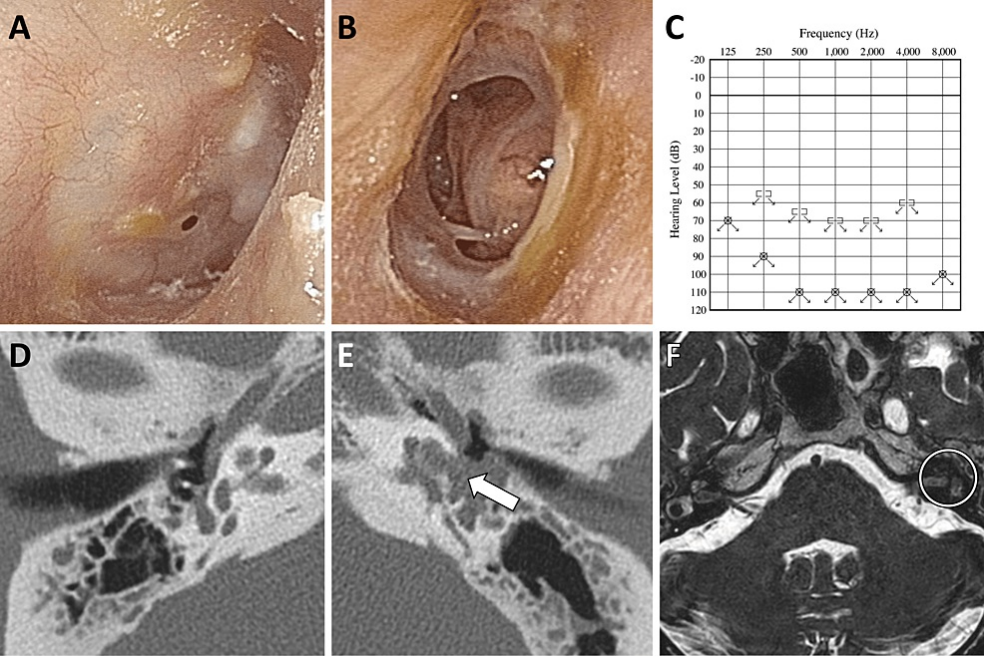
Preoperative clinical findings. (A) Endoscopic finding of the right tympanic membrane. (B) Endoscopic finding of the left tympanic membrane. (C) Pure tone audiogram. (D and E) Axial CT images of the right and left temporal bones, showing soft tissue density with osteolysis of the left cochlea (white arrow). (F) Heavy T2-weighted MR image showing reduced signal intensity in the left inner ear (white circle).

On initial examination, the right ear showed a pinhole perforation in the anteroinferior quadrant of the tympanic membrane, with no otorrhea (Figure 1A). The left tympanic membrane had a large perforation with otorrhea (Figure 1B). Pure tone audiometry revealed bilateral profound sensorineural hearing loss (bilateral scale-out) (Figure 1C). No nystagmus was observed with the positional and positioning nystagmus test using an infrared charge-coupled device camera. Video head impulse testing (vHIT) showed decreased vestibulo-ocular reflex (VOR) gain in all semicircular canals and catch-up saccades (CUS) in bilateral horizontal and posterior semicircular canals. Computed tomography (CT) showed only a small amount of soft tissue density in the right middle ear, but the left side showed a large amount of soft tissue density with osteolysis of the cochlea (Figure 1D-1E). The left inner ear signal intensity was highly reduced in the heavy T2-weighted magnetic resonance (MR) images, suggesting fibrosis on the left side of the cochlea (Figure 1F). Serum anti-neutrophil cytoplasmic antibody (ANCA) was negative, and there were no abnormal lung shadows on CT images, renal dysfunction, or skin symptoms suggestive of eosinophilic granulomatosis with polyangiitis (EGPA). It was determined that CI of the right ear was necessary as soon as possible because irreversible changes in the left cochlea were suggested.

A right CI and myringoplasty were performed simultaneously one and a half months after the patient's first visit. The patient was treated with systemic corticosteroids preoperatively to control middle ear inflammation. The corticosteroid used was prednisolone, administered at an initial dose of 20 mg and tapered over one week. No systemic corticosteroids were administered postoperatively.

**Figure 2:**
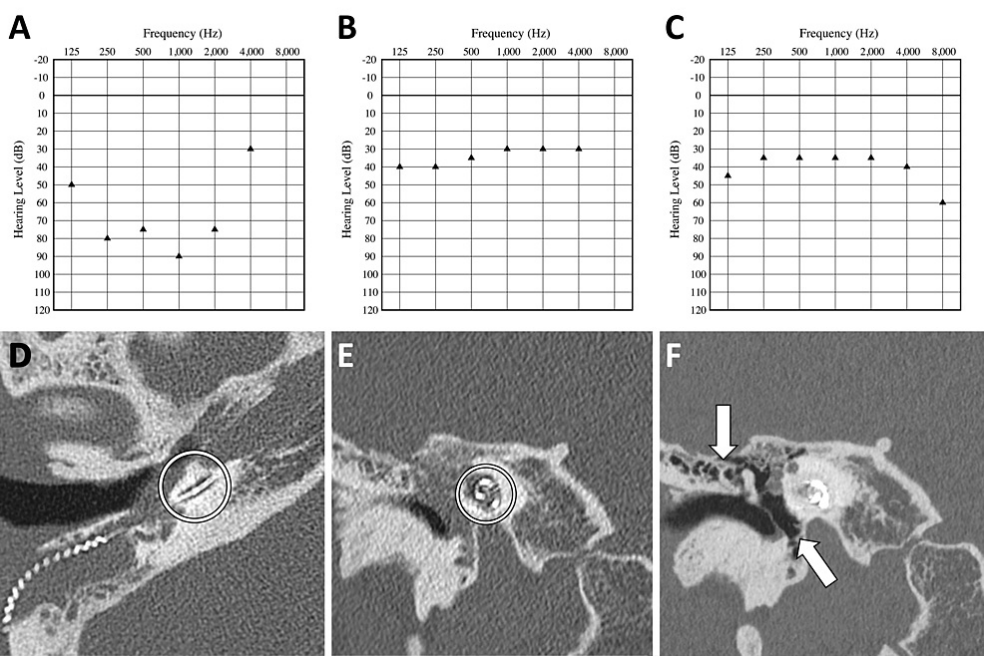
Postoperative clinical findings. (A) Hearing threshold result at five months after cochlear implantation (CI). (B) Hearing threshold result at seven months after CI (one month after introduction of dupilumab). (C) Hearing threshold results at 32 months after CI (25 months after introduction of dupilumab). (D and E) Axial and coronal CT images showing the electrodes correctly inserted into the right cochlea (white circle) and soft tissue density in the right middle ear. (F) Coronal CT image after introduction of dupilumab, showing that the soft tissue density in the right middle ear had disappeared (white arrow).

Intraoperatively, the round window was identified and opened, but no leakage of perilymph was found. When the cochleostomy was performed, the scala tympani was filled with granulation tissue. The device used was CI632 (Cochlear Ltd., Australia). The electrode was inserted smoothly despite the granulation tissue, and all electrodes were successfully placed. Intraoperative auto-neural response telemetry (NRT) did not produce responses from all electrodes, but responses were obtained in some channels when the stimulus and recording parameters were adjusted. Postoperative CT confirmed that the electrodes were correctly inserted into the cochlea (Figure 2D-2E). The perforation of the right tympanic membrane was closed, the otorrhea stopped, and there has been no re-perforation since.

Five months following surgery, the mean hearing threshold with the cochlear implant was poor at 67.5 dB (Figure 2A). However, when dupilumab was started due to worsening CRSwNP seven months after CI, the mean hearing threshold improved rapidly to 31.3 dB in the following month, despite no significant changes in the cochlear implant mapping (Figure 2B). Dupilumab was continued thereafter. At 32 months after CI and 25 months after the introduction of dupilumab, his mean hearing threshold was maintained at 36.3 dB (Figure 2C), and the soft tissue density in the right middle ear identified on postoperative CT had disappeared by this time (Figure 2F). Compared to the postoperative CT, the left temporal bone CT showed reduced soft tissue density in the left middle ear, but progression of ossification in the left inner ear. While his hearing threshold improved, his speech perception test score was not satisfactory at 10% (monosyllable), and he continues rehabilitation.

## Discussion

The diagnostic criteria for EOM were proposed by Iino et al. [1], and the disease concept is now widely accepted internationally. EOM patients have been reported to experience gradual or sudden deafness. Possible mechanisms include pathogen presence, inner ear damage from eosinophilic middle ear inflammation, and eosinophil-induced inner ear vasculitis [2]. In the present case, the patient presented with chronic otitis media featuring eosinophil-containing otorrhea, asthma, and nasal polyps. With no evidence of EGPA or hypereosinophilic syndrome, an EOM diagnosis was made. The primary treatment consisted of intratympanic corticosteroid ear drops. Despite this, the patient’s hearing progressively deteriorated, culminating in sudden bilateral deafness. The high fever and vertigo preceding the deafness might suggest possible labyrinthitis due to a systemic infection with some pathogen. Preoperative vestibular function tests indicated severe dysfunction of the semicircular canals as well as the cochlea. CT imaging revealed osteolysis around the left cochlea, potentially resulting from EOM-related and infection-induced labyrinthitis.

Recent studies suggest that biologics effective in severe asthma may also benefit EOM. These include omalizumab (anti-IgE antibody), mepolizumab (anti-IL5 antibody), benralizumab (anti-IL5 receptor antibody), and dupilumab (anti-IL4Rα antibody). While no official recommendations exist for biologic use in EOM, limited reports indicate dupilumab may be most effective among these agents [7]. Nakashima et al. conducted a study on 14 patients with concurrent EOM and refractory CRS or asthma treated with dupilumab [3]. They reported significant improvements in systemic corticosteroid use and severity scores in the dupilumab group compared to the non-treated group. Notably, all patients successfully discontinued systemic corticosteroid therapy after initiating dupilumab. Moreover, EOM severity and temporal bone CT findings showed significant improvement compared to pre-treatment.

Iino et al. evaluated the efficacy of dupilumab in three EOM patients with comorbid asthma [6]. These patients had previously failed to respond adequately to topical and systemic corticosteroids and other molecular-targeted therapies. Following dupilumab initiation, the authors observed dramatic decreases in disease severity and improved air-conduction hearing levels in all patients. Importantly, bone-conduction threshold deterioration appeared to be suppressed during dupilumab treatment in all three cases. Previously, surgical interventions such as tympanoplasty for EOM were contraindicated due to the risk of bone-conduction threshold deterioration [13]. However, Kikuchi et al. reported a case where tympanoplasty was successfully performed on an EOM patient in long-term remission with dupilumab, with a favorable postoperative course [14]. Additionally, they reported successful myringoplasty in EOM patients responding well to biologics including dupilumab [15]. Currently, surgical interventions, such as tympanoplasty and myringoplasty, are considered safe and potentially beneficial in EOM patients who demonstrate a good response to biological therapies.

CI in patients with EOM has been reported infrequently, with inconsistent hearing outcomes. Consequently, the optimal surgical technique and timing for CI in this population remain undetermined. Iwasaki et al. described a case of a 42-year-old woman who underwent CI seven years after developing bilateral deafness due to EOM at age 35 [8]. The left cochlea was ossified, necessitating CI in the right ear. A Nucleus 22 device (Cochlear Ltd., Australia) was used, with 25 electrodes reportedly inserted without difficulty, though detailed intraoperative findings were not provided. Six years post-CI, the patient demonstrated good speech perception, achieving a monosyllable test score of 32%. However, the postoperative course was complicated by bilateral middle ear effusion with gelatinous mucoid fluid. This complication required ventilation tube insertion and frequent administration of local and systemic corticosteroids.

Takahashi et al. reported two cases of EOM patients undergoing CI. In a 53-year-old female, CI was performed immediately due to the absence of otorrhea. Conversely, in a 65-year-old female, CI was delayed for three years because of persistent otorrhea [9]. Flex28 (MED-EL GmbH., Austria) was used for both patients. In the first patient, the electrode array was fully inserted via the round window approach. For the second patient, due to scala tympani ossification, the electrode array was inserted into the scala vestibule. Three years post-surgery, the first patient achieved an excellent speech perception test score of 92% (monosyllable), while the second patient had a poor score of 20%. Notably, both patients experienced postoperative tympanic membrane perforation and otorrhea, indicating that postoperative otorrhea does not necessarily correlate with poor hearing outcomes. These findings suggest that early CI may be considered even in cases of persistent preoperative otorrhea.

On the other hand, Kojima et al. reported a case series of CI for chronic otitis media, including one EOM patient who underwent CI using a subtotal petrosectomy (Rambo operation) [10]. This procedure involves radical mastoidectomy, eustachian tube closure, tympanomastoid obliteration with fat tissue, and blind sac closure of the external ear canal. The 44-year-old male patient had a CI24R (Cochlear Ltd., Australia) implanted in his left ear due to right cochlear ossification. No complications were observed during the two-year follow-up. Sugimoto et al. also reported CI in two EOM patients (64- and 71-year-old men) employing the same subtotal petrosectomy, achieving good postoperative hearing thresholds of 30-34 dB [11]. Similarly, Urabe et al. described a favorable outcome in a 65-year-old man with EOM using a similar surgical technique [12]. This patient, who had severe bronchial asthma, initially underwent rotating biologic therapy for EOM, cycling through omalizumab, mepolizumab, and dupilumab. However, due to worsening bronchial asthma, treatment was eventually returned to omalizumab. Despite increased middle ear granulation and persistent otorrhea, a CI622 (Cochlear Ltd., Australia) was implanted. Two years post-CI, the patient demonstrated good speech communication with a threshold of 30 dB and a speech perception test score of 58.3% (auditory).

Given the limited reports on the use of biological agents before and after CI in patients with EOM, it remains challenging to determine the optimal surgical approach, timing, and efficacy of biological agents for CI in EOM patients based on existing literature. However, as discussed below, our experience with the present case suggests that the administration of dupilumab may be beneficial in the CI treatment of EOM patients.

In the present case, CI was performed using a traditional approach. Otitis media did not worsen significantly enough to necessitate corticosteroid use until dupilumab was initiated after the surgery. However, without dupilumab, EOM inflammation might have eventually escalated, potentially requiring corticosteroid intervention, as reported by Iwasaki et al. The patient continued to have a high hearing threshold with the cochlear implant until the introduction of dupilumab. Intraoperative findings revealed no perilymph leakage when the round window was opened, and the basal turn of the cochlea was filled with granulation tissue, indicating severe EOM-related inflammation. Despite preoperative systemic corticosteroid administration, granulation tissue persisted in the cochlea. Postoperative CT findings confirmed correct electrode placement, ruling out insertion errors. CT findings at this time showed increased soft tissue density in the middle ear, suggesting worsening EOM. However, subsequent CT findings after the introduction of dupilumab showed resolution of this density, indicating effective EOM control by dupilumab.

Recent research has demonstrated the significant efficacy of dupilumab in treating granulation-type EOM [6]. Therefore, the improved hearing threshold with the cochlear implant after the introduction of dupilumab may be attributed to the reduction or resolution of the granulation in the cochlea caused by dupilumab, enabling more efficient electrical stimulation from the electrode to the spiral ganglion. The poor intraoperative NRT results might also be attributed to the presence of inflammatory granulation tissue within the cochlea, potentially interfering with effective stimulation of the spiral ganglion.

Despite improved hearing thresholds with the cochlear implant after the introduction of dupilumab, speech perception ability did not improve as significantly as previously reported. This outcome may be due to irreversible retrocochlear damage caused by strong inflammation during the process leading to deafness. Given that early dupilumab intervention may reduce bone-conduction threshold deterioration [6], prompt and aggressive dupilumab treatment could potentially prevent irreversible and severe hearing loss in EOM cases refractory to steroid therapy. For cases considering CI after deafness onset, adequate inflammation control with dupilumab may obviate the need for subtotal petrosectomy, allowing for standard CI procedures. 

The advent of biologics represents a potential paradigm shift in EOM treatment. As the EOM treatment landscape continues to evolve, careful monitoring of future trends is imperative.

## Conclusions

This case report demonstrates the potential efficacy of dupilumab in controlling middle and inner ear inflammation in patients with EOM undergoing CI. Our findings suggest that adequate control of inflammation with dupilumab before or after CI may contribute to improved postoperative hearing outcomes. However, as this is a single case observation, further research with larger patient cohorts is necessary to validate these results.
